# Fascicle localisation within peripheral nerves through evoked activity recordings: A comparison between electrical impedance tomography and multi-electrode arrays

**DOI:** 10.1016/j.jneumeth.2021.109140

**Published:** 2021-07-01

**Authors:** Enrico Ravagli, Svetlana Mastitskaya, Nicole Thompson, Elissa J. Welle, Cynthia A. Chestek, Kirill Aristovich, David Holder

**Affiliations:** a Medical Physics and Biomedical Engineering, University College London, UK; b Department of Biomedical Engineering, University of Michigan, Ann Arbor, MI, USA

**Keywords:** Peripheral nerves, Fascicular anatomy, Image reconstruction, Electrical impedance tomography, Multi-electrode arrays

## Abstract

**Background:**

The lack of understanding of fascicular organisation in peripheral nerves limits the potential of vagus nerve stimulation therapy. Two promising methods may be employed to identify the functional anatomy of fascicles within the nerve: fast neural electrical impedance tomography (EIT), and penetrating multi-electrode arrays (MEA). These could provide a means to image the compound action potential within fascicles in the nerve.

**New method:**

We compared the ability to localise fascicle activity between silicon shanks (SS) and carbon fibre (CF) multi-electrode arrays and fast neural EIT, with micro-computed tomography (MicroCT) as an independent reference. Fast neural EIT in peripheral nerves was only recently developed and MEA technology has been used only sparingly in nerves and not for source localisation. Assessment was performed in rat sciatic nerves while evoking neural activity in the tibial and peroneal fascicles.

**Results:**

Recorded compound action potentials were larger with CF compared to SS (∼700 μV vs ∼300 μV); however, background noise was greater (6.3 μV vs 1.7 μV) leading to lower SNR. Maximum spatial discrimination between Centres-of-Mass of fascicular activity was achieved by fast neural EIT (402 ± 30 μm) and CF MEA (414 ± 123 μm), with no statistical difference between MicroCT (625 ± 17 μm) and CF (*p* > 0.05) and between CF and EIT (*p* > 0.05). Compared to CF MEAs, SS MEAs had a lower discrimination power (103 ± 51 μm, *p* < 0.05).

**Comparison with existing methods:**

EIT and CF MEAs showed localisation power closest to MicroCT. Silicon MEAs adopted in this study failed to discriminate fascicle location. Re-design of probe geometry may improve results.

**Conclusions:**

Nerve EIT is an accurate tool for assessment of fascicular position within nerves. Accuracy of EIT and CF MEA is similar to the reference method. We give technical recommendations for performing multi-electrode recordings in nerves.

## Highlights

• Fascicle localization power of EIT and MEA was evaluated against MicroCT reference scans.
• Tested MEAs based on silicon shanks and carbon fibers in rat sciatic nerves.
• Fast neural EIT and multi-electrode arrays can detect evoked activity within the nerve.
• EIT and carbon fiber MEA have better source localization power than silicon MEA.
• Technical recommendations for insertion of MEAs into the nerves are provided.

## 1 Introduction

### 1.1 General context

Peripheral nerves comprise several bundles of nerve fibres (fascicles), each of them with a unique function. Groups of fascicles within the nerve are surrounded by layers of connective tissue. There are three layers of connective tissue that support nerve fibres and maintain the relative positions of neural elements – the endoneurium, epineurium and perineurium (Stewart, 2003). Detailed maps of peripheral nerve topography and fascicular organisation are required to allow the desired fascicles to be localised accurately for stimulation or recording with electrodes (Aristovich et al., 2019). There is still no definitive map of nerve fascicular anatomy in humans; most evidence suggests that nerve structure varies between individuals (Settell et al., 2020). Animal studies suggest there is somatotopic organisation in some nerves, i.e*.* each fascicle within the nerve carries nerve fibres which innervate a specific target – an organ or tissue – within the body (Bäumer et al., 2015; Stewart, 2003; Zill et al., 1980). Inter-individual variations of the functional anatomy of peripheral nerves require that the positioning of electrodes for selective functional stimulation should be adapted for each individual.

This is relevant to vagus nerve stimulation (VNS). The vagus nerve is a complex nerve innervating most visceral organs and consequently is an attractive target for neuromodulation. VNS has been shown to be effective in relieving drug-resistant epilepsy (Englot et al., 2011), depression (Carreno and Frazer, 2017), heart failure (De Ferrari et al., 2010), and chronic inflammatory disorders (Koopman et al., 2016). This procedure is accomplished by placing an electrode cuff around the right or left cervical vagus which results in general stimulation of both efferent and afferent fibres of the vagus nerve. This may result in significant side effects due to increased vagal outflow to off-target organs such as the larynx and GI system, which may cause hoarseness, dyspnoea, nausea, pain, anxiety, and cough (Ben-Menachem, 2001); which compromises the efficacy of VNS. In humans, the vagus nerve at the cervical level comprises 5 to 8 fascicles on average (individual variations of 1 to 21 fascicles per side; (Hammer et al., 2018; Verlinden et al., 2016)). Surprisingly, the functional anatomy of the fascicles is not known. It is unclear whether each fascicle contains only one type of fibre – afferent or efferent – or both, and if they are somatotopically arranged. To avoid off-target effects and improve the overall efficacy of VNS, the targeted stimulation of a select group or subset of fibres with known anatomical projection within the trunk of the vagus nerve is desirable (Thompson et al., 2019).

Currently, there is no technique allowing non-invasive imaging and tracing of organ-specific projections of the vagus nerve with fascicular resolution. Electrophysiological recordings of the evoked or spontaneous axonal traffic with the aid of peripheral nerve interfaces (PNI) could assist in the development of a map of functional anatomical organisation of the vagus nerve. However, the majority of PNI which permit intraneural, intrafascicular recordings are invasive or lack fascicular resolution.

Currently, only three methods allow electrical imaging of action potentials (AP): multi-electrode arrays (MEAs), inverse source analysis (ISA) and fast neural electrical impedance tomography (EIT). MEAs are electrophysiological probes with multiple recording sites arranged in a regular pattern. They passively record voltages and are mainly used for brain recordings, but some examples of use in nerves exist. ISA can reconstruct a spatial distribution of current density sources starting from passive voltage recordings and can be achieved from both invasive MEA electrodes and epineural cuff electrodes. Fast neural EIT with a nerve cuff was recently introduced for imaging evoked fascicular activity and has multiple advantages compared to ISA. EIT can be achieved with a nerve cuff and thus it is non-penetrating; however, MEAs have a better immediate signal-to-noise ratio (SNR) compared to EIT.

In this paper, we compare the feasibility of multi-electrode arrays and fast neural electrical impedance tomography as a tool for imaging local axonal traffic in peripheral nerves. The experiments hereby described are, as in previous studies by our group, focused on the rat sciatic nerve, which serves as a model and preliminary step to assess MEA technology before progressing to imaging the vagus nerve in larger animals in the future (e.g. pigs).

### 1.2 Fast neural electrical impedance tomography

Electrical impedance tomography is a non-invasive technique which allows imaging of the variation of electrical impedance inside an object of interest by reconstruction of impedance measurements collected from external electrodes (Holder, 2005). EIT has lower spatial resolution of ∼10% of the electrode array diameter compared to other types of tomographic techniques such as MRI or CT, but it allows for a much higher temporal resolution of milliseconds and might be performed with compact and relatively inexpensive hardware. As such, EIT can provide continuous or semi-continuous monitoring.

One of its biomedical applications is ‘fast neural EIT’ in which neuronal activity is imaged by the detection of small variations in electrical impedance produced by the opening of ion channels during firing. Opening of ion channels decreases membrane resistivity, reflected in a decrease in bulk resistivity of tissue of ∼0.1%. This can be imaged using EIT hardware together with averaging over repeated electrically evoked compound action potentials (CAPs) in nerve or physiologically evoked responses in brain. The process of averaging does not affect temporal resolution. This last step is necessary as the averaging process improves SNR and allows the neural impedance change signal to be detectable over noise.

Early studies reported neural impedance changes in humans (0.001% at 1 Hz with scalp electrodes) (Gilad and Holder, 2009), crab nerves (−0.2% at 125 and 175 Hz) (Oh et al., 2011), and rat somatosensory cortex (−0.07% at 225 Hz) (Oh et al., 2011), which suggested the possibility of performing imaging. Subsequently, fast neural EIT has been demonstrated in brain in both simulation (Aristovich et al., 2014) and experiments (Aristovich et al., 2016). More recently, fast neural EIT has been demonstrated as a method for imaging evoked compound activity in the rat sciatic nerve with a timescale of milliseconds (Aristovich et al., 2018; Ravagli et al., 2019, Ravagli et al., 2020) and an accuracy of ∼10% of the nerve diameter. Aristovich et al. reported a single-shot real-time SNR of 0.80 ± 0.03, which was increased ten times to 8.0 ± 0.3 with coherent averaging before image formation. Imaging of APs could also be achieved by ISA; however, EIT has multiple advantages: a potentially unique solution to the mathematical inverse problem; a number of independent measurements which is O(N^2^) compared to O(N), which means that a larger number of independent data can be obtained from the same number or electrodes; no dipole source cancellation problem, which means that sources of opposing polarity close together can still be correctly identified, and no theoretical limit on accuracy (Aristovich et al., 2018). Some of the potential disadvantages of EIT, in the context of neural engineering, are the presence of additional sources of noise given by spontaneous neural activity and the technical challenges related to real-time imaging, e.g. the need to drive multiple currents to avoid reduction of temporal resolution (Hope et al., 2019).

### 1.3 Multi-electrode neural probes

Peripheral nerve electrodes can be divided into three categories: nerve-surface electrodes, which include cuff electrodes and flat interface nerve electrodes (FINEs), penetrating electrodes such as longitudinal intra-fascicular electrodes (LIFEs), transverse intra-fascicular multichannel electrodes (TIMEs), Utah Slanted Electrode Arrays (USEAs), and regenerative electrodes (for a detailed review please see Rijnbeek et al. (2018)). Surface electrodes have been relatively successful in many studies but do not produce high levels of selectivity and fascicular resolution, whilst regenerative electrodes can potentially produce high selectivity but have limited applications requiring cutting and re-growth of the nerve through the electrode.

MEAs generally comprise a base with multiple extrusions that have electrode tips; however, designs may vary. The arrays are inserted into peripheral nerves so that there are multiple recording or stimulation sites. Depending on the number and distribution of neural contacts, and depending on probe design in general, the extent of the damage caused to the nerve by MEAs will vary.

FINEs can be used to reshape the nerve into a flat geometry, allowing an increase in surface area and the movement of fascicles closer to the surface. This type of electrode array was recently used in canine sciatic nerve to correlate neural recordings with muscle activity by means of a Bayesian signal processing approach (Eggers et al., 2018).

Cuff electrodes with high electrode count have recently been used to classify naturally evoked CAPs using spatiotemporal signatures (Koh et al., 2019); however, the use of multiple sets of spatially distanced ring arrays has the disadvantage of requiring implantation of a wider cuff.

LIFEs are inserted into individual nerve fascicles and lie parallel to the fibres inside. As LIFEs are inserted into the nerve, they are able to record from or stimulate a small group of fascicles, producing high selectivity (Rossini et al., 2010). However, due to the geometry of the electrode, at least one electrode per fascicle is required.

TIMEs are implanted into nerves transversely. They can therefore access multiple groups of nerve fibres and have a higher selectivity compared to LIFEs as they can record or stimulate different subsets of axons within a fascicle. In an acute study assessing the spatial selectivity of TIMEs, they were used to record neural activity in rat sciatic nerve in response to functional stimuli. It was only possible to discriminate stimuli coming from two different toes with a selectivity index of 71%. One of the main limiting factors was the partially overlapping receptive fields in the afferent fibres of peripheral nerves (Badia et al., 2016).

Another type of electrode array is the Utah Slanted Electrode Array. USEAs enabled the decoding of 13 different movements when implanted in the ulnar nerve of humans (Davis et al., 2016). These electrodes comprise 100 shafts in a 10 × 10 configuration with 400 μm spacing and 80 μm thickness. The tip of each shaft acts as an electrode and is sharpened. An optional slanted arrangement from 0.5 to 1.5 mm depth reduces the number of redundant electrodes and gives access to more fascicles (del Valle and Navarro, 2013). However, a chronic study in feline sciatic nerve showed that the USEA induced inflammation, scarring and rearrangement of fascicles, and that the recording qualities of the assay rapidly declined (Christensen et al., 2014). In some cases, there was also significant damage to the vasculature in the nerve – the source of bleeding after array explantation (Christensen et al., 2014).

A recently developed technology for multi-electrode neural recordings is carbon fibre (CF) microelectrodes. These comprise an extremely narrow carbon fibre body (<10 μm diameter) with external coating (e.g. parylene), which reduces both insertion damage and tissue scarring compared to conventional electrodes. In 2013, Guitchounts et al. (2013) reported the development of a 16-fibre array in a bundle configuration and its use for recording multi-unit activity in the brains of zebra finches. In 2015, Patel et al. (2015) reported the development of a 16-channel CF linear MEA and a method for its chronical implantation in rat brain. Work from the same group reported detection of unit activity more than one month after implantation and minimal glial scarring (Patel et al., 2016). In 2018, Gillis et al. (2018) reported detection of spontaneous multiunit activity and stimulation-evoked compound responses from a small peripheral nerve in zebra finches. More recently, Welle et al. (2020) reported modifications to the fabrication and coating techniques for CF electrodes which significantly improved recording yield in rat brain. Guitchounts and Cox (2020) reported the development of a CF array with expanded channel count and its use in recording from the rat visual cortex, and CF microelectrode arrays with 150–250 μm penetration depth have been used for recording spontaneous signals from the vagus nerve in rat (Jiman et al., 2020).

Recently, spontaneous neural activity was also recorded for the first time in human awake subjects with the ultrasound-guided insertion of microneurography needles (Ottaviani et al., 2020). However, this type of measurement only allows for collection of data at one single spatial site.

For the purposes of fascicle localisation within the nerve, the choice of recording electrode depends on the compromise between the size and geometry of the electrodes, resolution and biocompatibility. Large electrode arrays with many contacts will provide better physical stability, selectivity and resolution but are more difficult to implant and more likely to cause damage. Electrodes need to have a high level of selectivity – ideally, it should be possible to localise a specific fascicle in the nerve, but so far this has not been achieved with any design.

The SNR of recording APs has been reported as ∼7 for rat brain with silicon MEAs (Ward et al., 2009) and 2.0–8.3 for rat cervical vagus nerve with CF MEAs (Jiman et al., 2020).

It is unclear what spatial resolution of recorded APs may be expected with MEA recording in peripheral nerve; however, some guidance may be obtained by considering the underlying relevant physics and empirical spatial accuracy of recordings with MEAs in brain and nerve. A small inter-electrode distance of <20 μm may lead to multiple electrodes detecting the same APs (Franke et al., 2012). This study deals with MEA probes with inter-electrode distance >100 μm on the cross-section of the nerve, so this is unlikely to be relevant. However, the CF probes used in this study have a distance of 50 μm between multiple rows. In a recent study performed on rat vagus nerve with the same CF arrays, Jiman et al. (2020) reported that, in some instances, CF electrodes located in the same position on opposite rows recorded coinciding spikes with no time delay but with different amplitudes, suggesting that these spikes are generated from neurones located between these opposite carbon fibres.

Electrode size and shape are relevant as electrodes report the average voltage present at their uninsulated recording site (Nelson and Pouget, 2010). Thus, electrodes with larger surfaces will spatially integrate voltage over a larger area, but the nature of the recorded signal remains localised. The most important factor is the spatial distribution of the neural activity which creates the voltage distribution. Individual, spontaneous APs are extremely local phenomena and can only be recorded from an electrode very close to the source of the AP and similar in size. For example, an electrode of diameter 10–20 μm might record spontaneous activity from one or a few brain neurones at a distance of <20 μm. In the brain, the maximum distance between source and recording site has also been investigated for local field potentials (LFPs) (Buzsáki et al., 2012; Einevoll et al., 2013). LFPs in the brain originate from volume conduction in the extracellular space of signals by multiple local sources, and their real “locality” has been heavily debated (Kajikawa and Schroeder, 2011; Herreras, 2016), with estimates ranging from ∼200 μm to several millimetres (Kajikawa and Schroeder, 2011). The unclear biophysical nature of the LFPs and the physiological differences between brain and nerve preclude the possibility of directly translating LFP studies to nerve. However, a wide spatial distribution of voltage (e.g. hundreds of μm) in nerve can be expected from CAPs, as they share some similarities with LFPs, specifically the activation of multiple current sources all near each other, with the extracellular space acting as a signal integrator.

Temporal resolution in MEA recordings is determined mainly by recording bandwidth and sampling rate, as the involved phenomena do not exceed spectral content of a few KHz. LFPs are usually measured at low frequencies (<300 Hz) (Bozer et al., 2017; Obien et al., 2015) and spontaneous APs are measured in the 300–3000 Hz bandwidth (Obien et al., 2015). A temporal resolution 1 ms or lower is thus usually achieved.

### 1.4 Purpose

The main purpose of this study was to compare fascicle localisation power across the cross-section of the nerve between invasive multi-electrode probes and fast neural EIT. We tested two MEAs, based on silicon shanks (SS) or carbon fibres, and nerve EIT and compared them against reference MicroCT images of nerve fascicular anatomy. We addressed the following questions:

a) Is it possible to record fascicular evoked compound action potentials (CAPs) with multi-electrode probes in rat sciatic peripheral nerve, together with EIT?
b) Which methodological details are important for proper insertion of multi-electrode probes in the rat sciatic nerve?
c) How does source localisation on the nerve cross-section compare between the invasive multi-electrode probes and fast neural EIT? Which technique gives the highest spatial separation between fascicles?

A typical rat sciatic nerve is 1400 μm in diameter, with two predominant fascicles occupying approximately 50% of the space each. Considering that these fascicles are not perfectly elliptical, equally sized, and do not sit right on the edge of the nerve, the best possible source peak separation result that can be reached by EIT and MEA is approximately 600 μm.

## 2 Methods

### 2.1 Experimental design

The two main branches of the rat sciatic nerve (tibial and peroneal) were stimulated electrically to evoke spatially separated (fascicular) CAPs. At the level of common sciatic nerve, the evoked fascicular traffic was recorded with an EIT cuff electrode and SS and CF probes. The SS probe consisted of 4 shanks with 4 recording sites each, and thus electrodes in this probe were arranged in a 4 × 4 configuration. The CF probe consisted of 16 CF electrodes arranged in an 8 × 2 configuration with fixed depth.

**Fig. 1:**
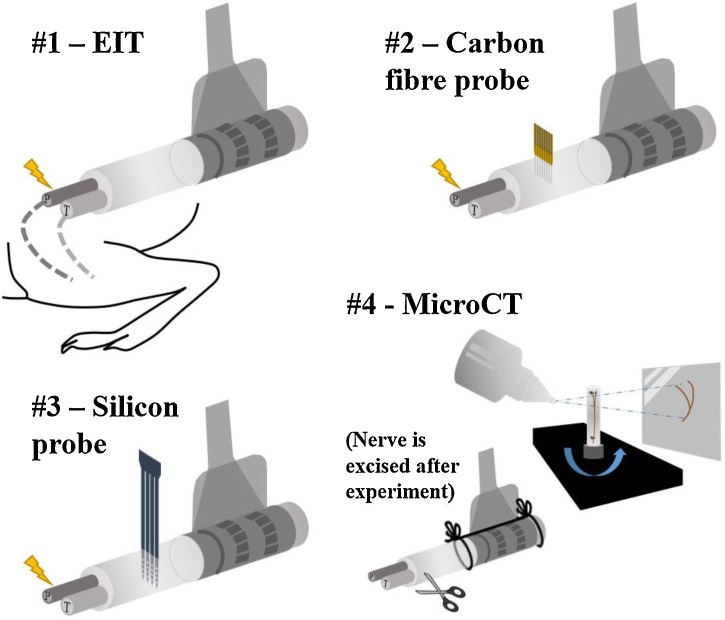
Experimental procedure. Neural activity was evoked on individual tibial (T) and peroneal (P) fascicles while performing EIT (#1), then again while recording from CF (#2) and from SS (#3) probes inserted close to the EIT cuff. Following the conclusion of the electrophysiological recordings, the nerve was excised, stained and then underwent MicroCT imaging (#4).

Recording methods were tested sequentially in order of increasing invasiveness (EIT, CF, and SS) (Fig. 1):

1. As a first step, fast neural EIT recordings of evoked fascicular activity were performed.
2. Subsequently, the CF probe was inserted perpendicularly to the nerve's length and recordings of evoked fascicular activity were performed. The EIT electrode cuff was left on the nerve to provide mechanical stability and a landmark reference for the MEA recordings. At this stage, recording healthy CAPs with the CF probe was considered evidence that the nerve received no significant damage and a reason for continuing the experiment.
3. The CF probe was removed and replaced with the SS probe in approximately the same position. Then, recordings were performed as in the previous step.
4. After the conclusion of the experiment, the nerve was excised and MicroCT-scanned. This provided the reference gold standard for the location of fascicles inside the nerve and allowed assessment of possible nerve damage.

Choice of probe geometry, which is described in more detail below, was mainly directed by availability from the manufacturers. The ideal configuration would have been a square grid of electrodes placed at regular distance along the cross-section of the nerve, with electrode supporting structures sufficiently thin to allow perfect insertion. Alternatively, a 3D structure allowing penetration at different depths like the USEA probes would have been acceptable. The SS probe fit the square grid requirement, but the width and thickness of the shanks made insertion challenging. We considered rotating 90° and stacking multiple SS probes to obtain a 3D grid of electrodes; however, manufacturing limits would have left at least 0.5 mm separation between the shanks’ planes, making insertion in the rat sciatic nerve impossible. The CF probe had the main drawback of fixed depth for all carbon shanks, which only allowed spatial sampling along the lateral direction of the nerve's cross-section. Cutting a single CF probe in a slanted profile was an option made available by the manufacturer and investigated preliminarily; however, stacking multiple planes of slanted fibres was beyond our current technical capabilities and would have made insertion more difficult due to bending/buckling beyond the first deeper row of fibres.

Evaluation of fascicle localisation power for each technique was achieved by computing the Centre-of-Mass (CoM) over the nerve cross-section at peak activation for each recording and then evaluating the distance on the nerve's cross-section between the tibial and peroneal CoMs. We expected higher distance for techniques with better discrimination power, up to maximum detected distance for the reference technique of MicroCT. Direct comparison between techniques was made problematic by the fixed depth of the CF probe which only allowed CoM computation along the horizontal direction. We addressed this problem by upscaling the distance metric estimated from carbon fibres data by a geometrical factor of √2, corresponding to adding one dimension to the CoM.

In healthy rat sciatic nerves not subjected to extensive mechanical manipulation, the internal position of each fascicle type (e.g. tibial) over the cross-sectional plane could be expected to be roughly similar due to the consistent functional anatomy of this type of nerve across animals (Badia et al., 2010, Badia et al., 2016). As such, CoM values for each fascicle group would show up in clusters and a scatter metric could be defined to quantify natural variability of CoM position. However, a clusterization procedure was necessary in this study to compensate for the effect of mechanical deformation and torsion caused by performing surgery, applying collagenase, and inserting probes into each nerve.

As a last step of our dataset analysis, we performed a qualitative comparison between predicted and achieved results for EIT and MEA by overlaying data from each nerve and generating average images over the nerve's cross-section for each fascicle.

### 2.2 In vivo preparation

Experiments were performed on sciatic nerves from adult male Sprague-Dawley rats weighing 400–550 g, with the same procedures as in (Aristovich et al., 2018; Ravagli et al., 2019, 2020). All animal experiments undertaken in this study were approved by the UK Home Office and in accordance with its regulations.

**Fig. 2:**
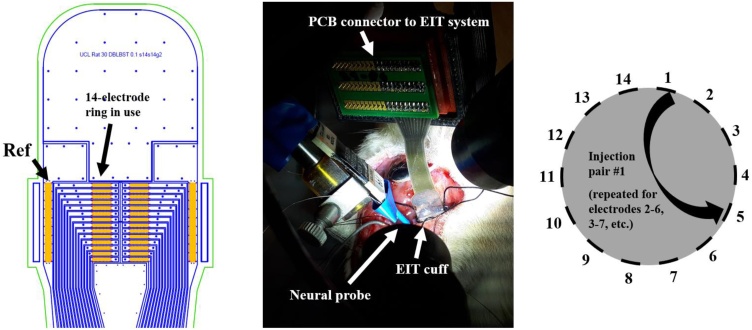
Left: AutoCAD drawing of the nerve cuff with electrodes (orange), outline of stainless-steel tracks (blue) and outline of external cuff boundary (green). Arrows and labels indicate the ring array and reference electrode used in our experiments. Middle: nerve cuff in an experimental setting, wrapped around the rat sciatic nerve, with the neural probe close by. Right: simplified representation of the cross-section of the nerve with EIT electrodes and example of one current injection pair.

Animals were anesthetised with urethane (1.3 g/kg, i.p.), intubated and artificially ventilated using a Harvard Apparatus Inspira Ventilator (Harvard Apparatus, Ltd, UK) with a 50/50% gas mixture of O2 and air. Electrocardiogram and respiratory parameters (respiratory rate, end tidal CO2) were monitored (Cardiocap 5, Datex Ohmeda). The core body temperature of the animal was controlled with a homeothermic heating unit (Harvard Apparatus, Kent, UK) and maintained at 37 °C. The animal was positioned prone, and the common sciatic nerve and its branches were dissected (Aristovich et al., 2018; Ravagli et al., 2019, 2020). The EIT cuff was placed around the main trunk of the sciatic nerve with the cuff opening facing superiorly (Fig. 2) and stimulation cuff electrodes (CorTec Gmbh, Freiburg, Germany) coated with PEDOT:pTS were placed around tibial and peroneal branches at ∼1–1.5 cm distally from the EIT cuff. Impedance of the Cortec cuffs post-coating was <1 KΩ, measured at the frequency of interest for this study, 9 kHz. A new EIT cuff was used for each experiment. The surgical preparation from the moment of anaesthesia onset to the beginning of recordings took on average 1 h. To avoid movement artefacts during recordings, neuromuscular blocking agent, pancuronium bromide (0.5 mg/kg, i.m.) was used.

After the EIT measurements of evoked fascicular traffic were completed (Sections 2.3–2.4.1), the surgical area was prepared for MEA recordings. The nerve was covered by dense epineurium; therefore, insertion of the thin fragile intraneural probes like CF and SS MEAs was challenging. To enable insertion of the MEAs, the nerve epineurium was treated with collagenase (10 mg/mL) at room temperature for 10 min (Type 4 collagenase, Worthington, NJ; Chen et al., 2017). A cotton pad (5 mm in diameter) was soaked in collagenase solution and placed on the surface of the nerve in the area of MEA probe insertion. After 10 min, the area was gently washed with another cotton pad soaked in saline. This procedure ensured local dissociation of collagen fibres present in the epineurium while leaving the nerve fibres intact. Still, there was some tissue resistance to intraneural insertion of MEAs due to the incomplete digestion of perineurium (connective tissue surrounding each fascicle). The epineurium was gently teased apart using fine forceps to avoid damage to the underlying nerve fascicles. The MEA probe was inserted transversely 2–3 mm distal to the EIT cuff (Fig. 2) with a micromanipulator (SM-15; Narishige International Ltd., London, UK). To facilitate the insertion of the probe, the nerve was stabilised by a gentle horizontal stretching with the help of a loose suture placed around the distal part of the nerve. First, recordings were performed with the CF MEA, followed by recordings with the SS MEA. The insertion of the CF MEA down to half-width of the nerve cross-section took approximately 30 min (including the tissue digestion procedure). The insertion of the SS MEA was even more challenging because of the wider and thicker shanks (100 × 50 μm compared to ∼8 μm CF electrodes). Tips of the SS probes were successfully inserted in most cases, but deeper progression of the probe inside the nerve was compromised, which is evident from CAP intensity values recorded with these probes (Section 3.2).

CFs were fragile and on occasion fractured and became unsuitable for use. All recordings presented here are with the majority of fibres (>80%) in the probe functional. Faulty fibres were removed from analysis as explained in the Methods section relative to MEA technical details. A new CF MEA was used for each experiment.

Following withdrawal of the CF MEA, the nerve was inspected visually through the optics system of the micromanipulator. SS MEA recording were only undertaken in the absence of any apparent nerve damage as evaluated by:

• Visual inspection not showing damage.
• CAPs recorded with the CF probe >50 μV.

At the end of the experiment, the animal was euthanised with an overdose of sodium pentobarbital (200 mg/kg), the position of the EIT cuff was labelled with the 6.0 cotton suture glued to the epineurium and marking the cuff opening for the subsequent cross-validation procedure. The sciatic nerve was excised together with main branches, fixed in 10% neutral buffered formalin and processed for MicroCT.

### 2.3 Evoked electrophysiological activity

CAPs and impedance changes were evoked in fast myelinated (A-beta/delta) sensory/motor fibres in the tibial and peroneal fascicles of the sciatic nerve by supramaximal biphasic pulse stimulation of individual branches at 2 mA amplitude, 20 Hz frequency and 50 μs pulse width with CorTec cuff tunnel bi-polar electrodes (CorTec GmbH, Germany) placed ∼1–1.5 cm distally from the EIT cuff. The current of 2 mA was chosen to maximize the magnitude ratio between the CAP and the stimulus artefact measured on the EIT cuff electrode array prior to EIT recordings. CAPs were recorded for a time of ≥1 min each.

### 2.4 Fast neural electrical impedance tomography

#### 2.4.1 Experimental setup and measurement procedure

EIT measurements were performed with a ScouseTom system (Avery et al., 2017), a recently developed high-performance EIT system composed of open-source hardware and software modules combined with a commercially available current source (model no. 6221, Keithley UK) and EEG amplifier (ActiChamp EEG amplifier, BrainProducts GmbH, Germany). The rat sciatic nerve cuff used in the experiments for recording surface CAPs and EIT data comprised two arrays of 14 electrodes, each one arranged in a circumferential ring, with two reference electrodes placed at the extremities of the cuff (Fig. 2, left panel) (Ravagli et al., 2019, 2020). Only one ring of electrodes was used for transversal (i.e. cross-sectional) current injections. The cuff was designed to wrap around a nerve with nominal 1.4 mm diameter. It was made from silicone rubber spun onto stainless steel foil 12.5 μm thick, coated with PEDOT:pTS (Chapman et al., 2019). Impedance of the electrodes post-coating was ∼1 kΩ, measured at the frequency of interest for this study, 9 kHz.

The EIT protocol comprised 14 transversal current injections with a skip-4 spacing drive pattern, which corresponded to ∼100°, previously identified by our group as one of the optimal protocols for this application in terms of resolution (Ravagli et al., 2019, 2020). Current was injected at 9 kHz and 60 μA. Frequency was increased from the value of 6 kHz used in our previous work to 9 kHz. This was in order to increase the difference between the EIT signal carrier and harmonics of the stimulation pulses which appear at lower frequencies, hence resulting in a demodulated EIT signal with less artefact contamination. Each current injection was 15 s long, leading to 300 repeated stimulation pulses at 20 Hz; the total protocol lasted 3.5 min.

#### 2.4.2 Post processing and image reconstruction

Raw impedance signals from experimental recording were demodulated to voltage changes over time “δV” by the Hilbert transform to yield the modulus with a ±2 kHz bandwidth around the 9 kHz EIT carrier. Demodulated δV traces were averaged over all the 300 repeated stimulation pulses to reduce noise and reach a SNR sufficiently high for successful EIT imaging. Some of the collected impedance traces were excluded from the reconstruction process based on the following criteria:

• Traces collected on faulty electrode. Faulty electrodes were defined as outliers in terms of either:
  ∘ background noise larger than 3 times the median of the array.
  ∘ amplitude of the demodulated signal being constant regardless of choice of injecting electrodes
• DC saturation of raw signal, defined as the signal being within 10% of the voltage range maximum of the EEG amplifier (±400 mV).
• δV background noise >3 μV.

Image reconstruction was performed as follows (Aristovich et al., 2018; Ravagli et al., 2019, 2020):

• The UCL PEITS fast parallel forward solver (Jehl et al., 2015) was used to compute solution to the EIT forward problem according to the complete electrode model (CEM). In the solver, injected EIT current was set to 60 μA and contact impedance of the electrodes was set to 1 kΩ. Rat sciatic nerve model geometry and mesh features are the same as in (Ravagli et al., 2019, 2020). The mesh had 2.63 M tetrahedral elements, with a maximum element size of 20 μm on the electrodes, 40 μm for the inner nerve region (under and between the electrodes), 60 μm for the outer part of the nerve region, and 420 μm for the external subdomain. Mesh quality was higher than 0.7 for >99% of the elements. Minimum mesh element quality was 0.47.
• Images were reconstructed by projecting the Jacobian matrix J obtained from the forward solution over a coarse hexahedral mesh (∼75 K elements, voxel size of 40 μm). Inversion of the coarse Jacobian matrix was done by 0th-order Tikhonov regularisation and noise-based voxel correction (Ventouras et al., 2000; Aristovich et al., 2018). Only the relevant fraction of the hexahedral mesh, namely the part of the nerve subdomain including the injection/measurement rings, was reconstructed.
• Noise-based image correction was performed by projecting random white noise into the voxel mesh with the same reconstruction parameters. In every reconstruction, intensity values for each voxel were divided by standard deviation of projected noise, resulting in a z-score of the conductivity perturbation with respect to background noise.
• Image post-processing was performed by median filtering (1-voxel radius) and mean filtering (3-voxel radius). Following post-processing, computation of CoM was performed for each fascicle over the dataset of EIT images thresholded at Full-Width Half-Maximum (FWHM).

**Fig. 3:**
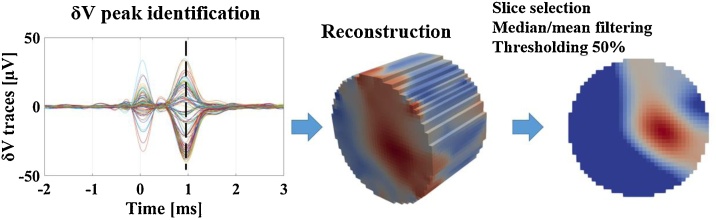
Left: Typical demodulated EIT traces from multiple injection pairs and recording electrodes grouped together. Traces show stimulation artefact at *T* = 0 and a later peak due to CAP-related conductivity changes. Middle: reconstructed conductivity changes in the voxel mesh. Right: final EIT image after slice selection and post-processing.

EIT reconstructions were evaluated at the time of peak average δV variation for each recording. Visualisation of reconstructed images was performed with Paraview (Kitware, New Mexico, USA). This process is summarised in Fig. 3.

### 2.5 Multi-electrode arrays

**Fig. 4:**
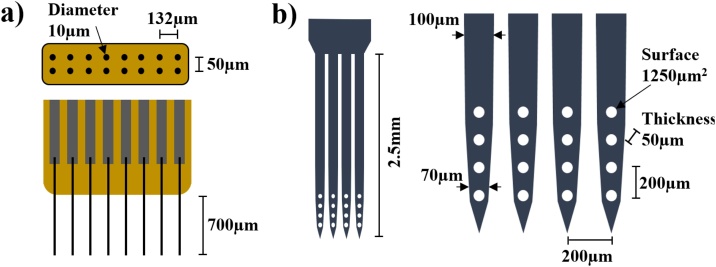
Left: top and side view of the Flex Array CF MEA. Right: side view of the SS MEA with zoom-in on the recording sites.

The CF MEAs were the FlexArray v3, which were made available from Chestek Lab within the NeuroNex MINT Hub (https://chestekresearch.engin.umich.edu/, https://mint.engin.umich.edu/). Each comprised 16 CF electrodes arranged in an 8 × 2 configuration, with a 132 μm pitch between the electrodes and 50 μm pitch between rows (Fig. 4a). Fibres were cut to a 700 μm exposed length in order to reach the centre of the nerve, which was ∼1.4 mm in diameter. Fibre diameter was ∼8 μm. The recording site, the tip of each fibre, was sharpened by blowtorch and coated with PEDOT (Welle et al., 2019). Impedance of the recording sites varied within a range of 10–60 kΩ, measured at 1 kHz by the manufacturer.

SS MEAs used in our experiments were a customized variant of a commercial design (NeuroNexus, Michigan, USA). Each probe comprised 4 depth shanks, 50 μm thick, with 4 recording sites each, producing a 4 × 4 matrix configuration. Vertical and horizontal spacing between recording sites was 200 μm, so the resulting grid covered a 600 × 600 μm area (Fig. 4b). The width of the shanks was ∼100 μm along the main shaft and ∼70 μm close to the tip. Each shank had a total depth of 2.5 mm (base to tip), which allowed a margin for inserting the recording sites deep inside the nerve. Recording sites had a surface area of 1250 μm^2^. Impedance varied from 0.3 to 0.6 MΩ, measured at 1 kHz by the manufacturer.

Both types of probes were subject to tip-sharpening processes by the manufacturers.

For both types of probes, the voltages were sampled at 50 kHz by connecting the probe to the same actiCHamp EEG amplifier used for EIT recordings through unitary gain analogue headstages (Plexon, TX, USA). A bandwidth of 15 kHz was used, given by the internal anti-aliasing filter of the recording system. Measurements were taken with respect to the circular reference ring present on the EIT cuff.

### 2.6 Reference imaging of fascicles by MicroCT

Reference images of nerve transversal sections with fascicles clearly visible were collected by MicroCT imaging to obtain fascicle ground truth locations in the form of CoM coordinates (Thompson et al., 2020; Ravagli et al., 2019, 2020).

Upon completion of the experiment, the EIT cuff was removed and the location of the cuff opening was marked with surgical suture. The nerve was then explanted, fixed in formalin, stained with 1% iodine solution, and scanned with a MicroCT scanner (Nikon XT H 225: molybdenum target, 4 W power, 3176 projections, 4 μm resolution, 35 kVp energy, 114 μA current, and 4 s exposure time). Scans were reconstructed with Nikon CT Pro 3D software and exported to MATLAB (vR2018b, MathWorks, Natick, USA).

MicroCT reference fascicle CoMs were evaluated with a custom MATLAB script by fitting the nerve external boundary to a circular profile after performing uniaxial deformation of the image. Co-registration between the MicroCT scans, EIT images, and MEA probe data was performed using the location of the cuff opening mark (surgical suture) which was visible in the MicroCT scans (Ravagli et al., 2019, 2020).

MicroCT scans were also evaluated visually by an expert operator to check for tissue damage.

### 2.7 Data analysis and statistics

*N* = 5 sciatic nerves from the left and right posterior legs of 3 animals were analysed. The right sciatic nerve from the first animal was not analysed since the first proof-of-concept recordings on the left leg lasted for an extended period and the experiment had to be terminated due to deterioration of physiological parameters.

**Fig. 5:**
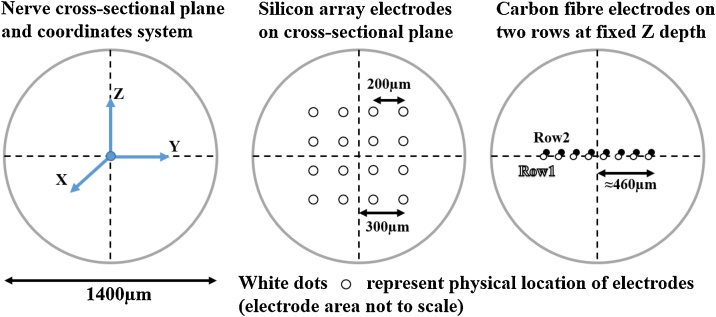
Left: orientation of the axes of the coordinate system on the cross-section of the nerve. Middle: position of electrodes from the SS MEA assuming perfect placement. Right: position of electrodes (tips) from the CF MEA assuming perfect placement. Both rows of CF electrodes have the same depth of *Z* = 0 and lay on the horizontal (X,Y) plane.

Diagrams below are oriented so that the X, Y and Z axes are respectively axial, horizontal and vertical in the nerve cross-section (Fig. 5).

**Fig. 6:**
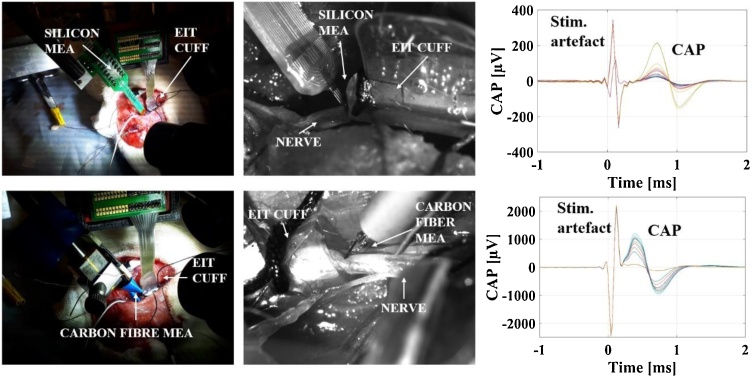
Upper panels: setup of EIT cuff and SS MEA probes around the rat sciatic nerve, with images of the probe being inserted, and representative CAP from stimulation of fascicle. Lower panels: same images and representative CAP for the CF MEA probe.

The main purpose of our work was to compare fascicle localisation power across the cross-section of the nerve for EIT and MEA probes. The nerve cross-section is represented by the (Y, Z) plane in Fig. 5. Both EIT and the SS MEAs return information which lays on this plane. EIT data consists of a conductivity image with 40 μm pixel resolution of the nerve cross-section while data from SS probe consists of a 4 × 4 matrix of recorded voltage as shown in Fig. 5, middle. The CF electrodes all have the same fixed depth (700 μm) and thus only record information in the middle of the nerve (Fig. 5).

For EIT, noise and SNR were computed and averaged over all the recorded δV traces for each fascicle. SNR was computed at the time of peak impedance change. The CoM for reconstructed EIT images was computed after FWHM thresholding (2.4.2) according to Equation (1):

(1) $$CoM(t_{Peak},y,z)=\frac{\sum_{i=1}^{N_{E}}d\sigma_{i}(t_{Peak})\cdot P_{i}(y,z)}{\sum_{i=1}^{N_{E}}d\sigma_{i}(t_{Peak})}$$

where dσ_i_(tPeak) is the post-FWHM conductivity variation amplitude recorded at peak time from the i-th element of the EIT image, whose position in the coordinate system is represented by Pi (x, y, z).

For both types of MEA probes, noise and SNR were computed from the peak time of evoked CAPs. The averaged CAP from all recording sites was used for this purpose. The CoM of fascicle activation was computed from MEA recording sites by evaluating the CAP data at peak time (positive peak) according to Equation (2):

(2) $$CoM(t_{Peak},x,y,z)=\frac{\sum_{i=1}^{N_{R}}V_{i}(t_{Peak})\cdot P_{i}(x,y,z)}{\sum_{i=1}^{N_{R}}V_{i}(t_{Peak})}$$

where V_i_(t_Peak_) is the voltage recorded at peak time from the i-th recording site, whose position in the coordinate system is represented by P_i_ (x, y, z). No FWHM thresholding was applied to MEA CAP recordings. We assume (as an approximation) that the coordinates of the MEA probes after insertion were aligned with the coordinate system centred on the nerve as in Fig. 5. As an example, the centre of the 4 × 4 grid of recording sites on the SS MEAs would match coordinates (0,0,0), assuming perfect insertion of the probes in the middle of the nerve cross-section. For all the above purposes, in recordings where stimulation artefact overlapped with the positive peak of the CAP due to short distance between the stimulating electrodes and MEA recording sites, the negative peak of the CAP was used in place of the positive peak.

Traces from damaged or non-contact recording sites were excluded from computation of the above features. Faulty electrodes were identified by either direct detection of a broken/non-contact fibre from microscope observation after insertion, or by analysis of background noise in recorded data. In this second case, electrodes with background noise more than double the median of the array were identified as outliers and removed.

Each of the measurements described above (EIT, CAPs on MEA arrays) was performed twice on each nerve and fascicle: twice for the tibial fascicle and twice for the peroneal. Final SNR and CoM values were averaged between the two repetitions for each technique and fascicle. Significance of peak δV traces and CAPs recorded from MEAs was determined in respect to background noise by paired t-test.

The ability of each technique to successfully discriminate the position of the two fascicles was evaluated by:

1. Computing the distance between tibial and peroneal fascicle CoM on the cross-sectional Y-Z plane, for each nerve and for each technique. This operation constituted a “within-subject” comparison of CoM location on the same nerve among different techniques.
2. Performing repeated measures ANOVA on the distance metric with different techniques as the within-subject factor, followed by paired T-tests among techniques.
3. Performing individual t-tests for each technique on the distance metric to evaluate statistical significance against the null hypothesis. In this test, the value of the distance metric for each technique is compared against zero. If the distance metric is found to have no significant difference from zero, the implication is that no significant separation between tibial and peroneal sources is detected.

Distance for CF data was computed as the absolute difference of tibial and peroneal CoM positions on the Y-axis. Due to the fixed depth of the electrodes, Z-axis information, i.e. the information along the vertical axis of the nerve's cross-section, was not available for this type of probe. For this reason, and with the aim to compare with EIT and SS probe results, we assumed the average angular position of 45° for real CoM locations and increased CF distance by a factor of square root of 2 (*∼*1.41).

The clusterization procedure for computing the scatter metric was performed as follows: for each technique, a rigid rotation was applied to each set of tibial/peroneal CoM coordinates. Scattering of fascicle clusters was quantified by taking standard deviation around mean fascicle CoMs, along both coordinate axes, for each fascicle. For each set of tibial/peroneal CoM coordinates belonging to a different nerve, a different rigid rotation angle was chosen to minimize the overall scatter of fascicle CoM clusters over the entire dataset. Post-clusterization scatter metric gives a numerical indication of the variability of CoM location from the same fascicle type after nerves are rotated to have maximum possible overlap, i.e. it corresponds to a measure of how spread out the cloud of CoM points is for tibial and peroneal. Residual scatter after clusterization procedure can be ascribed to physiological variability and is reported in the results.

Generations of images for comparison of results with predicted outcomes was performed as follows: for EIT, predicted outcome is shown as a graphical representation of the activated area over the cross-section of the nerve. Our previous studies in nerve EIT showed an area of activation qualitatively corresponding to ∼30–50% of the rat sciatic nerve diameter for evoked fascicular activity (Aristovich et al., 2018; Ravagli et al., 2019, 2020). Images of average EIT results from this study were generated by rotating reconstructed images for each nerve according to the angles identified in the clusterization analysis, and then performing pixel-by-pixel averaging. The expected spatial voltage distribution was the same for both MEA probes and was computed by simulating fascicular evoked activity in a Finite-Element Method (FEM) model of the nerve. Data from SS and CF MEA was subject to an upscaling procedure to achieve sufficient resolution for meaningful overlapping of images. MEA-SS recordings were upscaled from the native 4 × 4 resolution by resampling to double the resolution and applying a smoothing filter with a 3 × 3 kernel. This process was repeated iteratively 7 times. Resulting images for each nerve were rescaled to [0–1] intensity range and averaged together pixel by pixel. Average image was smoothed with a Gaussian filter with SD = 2 pixels and cropped to remove edge artifacts. MEA-CF recordings were subject to a one-dimensional (1D) version of the same procedure. For each technique, a representative 1D profile across the average image was chosen and reported along with standard error (SE) to show variation of intensity while traversing the nerve's cross-section.

All numerical values are reported as mean ± 1 SE unless specified.

## 3 Results

### 3.1 Electrophysiological data and technical considerations

It was possible to identify significant δV/voltage traces in all CAP/EIT recordings, as indicated by significant (p < 0.05) post-averaging peak variations from background noise level in response to evoked activity. The SNR was highest for SS MEAs, then CF MEAs, then EIT (∼400, 200 and 55, respectively) (Table 1). Data collected from CAP negative peaks amount to 20% of the dataset for both CF and SS recordings.

**Table 1:** Values of electrophysiological markers for each technique averaged over the entire dataset. Fascicles are marked as T/P for Tibial/Peroneal. All values are reported after averaging over repeated stimulation pulses.

	Electrophysiology markers – average values				
	Fascicle	# of δV traces (EIT) and voltage traces (MEA)	δV/CAP (μV)	Background noise (μV)	SNR (–)
EIT	T	109.6 ± 4.1	37.8 ± 12.1	0.6 ± 0.03	65.9 ± 24.1
	P		26.0 ± 4.8		44.8 ± 8.6
SS MEA	T	14.2 ± 0.1	425.7 ± 117.1	1.7 ± 0.5	434.7 ± 216.2
	P		261.4 ± 107.6		427.0 ± 285.5
CF MEA	T	15.3 ± 0.1	748.0 ± 257.6	6.3 ± 1.1	207.9 ± 101.6
	P		723.2 ± 315.3		209.6 ± 111.6

### 3.2 Fascicle discrimination power of different techniques

**Fig. 7:**
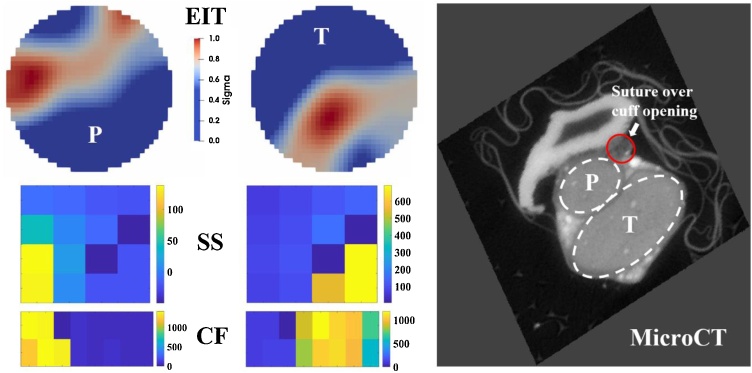
Example of results collected from the same nerve with different techniques (not to scale). Different fascicles are marked as T for tibial and P for peroneal. Left upper row: EIT reconstructions. Left middle row: CAP intensity values at peak time for the SS array. Left bottom row: CAP intensity values at peak time for the CF array. CAP values are expressed in μV. Right: MicroCT image of the nerve cross-section in the immediate vicinity of the EIT cuff. Fascicles (P and T) and suture marking the cuff opening are clearly visible.

It was possible to discriminate two unimodal peaks in EIT images which corresponded to the tibial (T) and peroneal (P) fascicles in all 5 nerves (example in Fig. 7, full dataset in Suppl. Material). EIT could successfully discriminate the positions of the tibial (T) and peroneal (P) fascicles. Both probes achieved a high degree of spatial discrimination of fascicle activity in the lateral direction. For the SS MEA, the highest recorded amplitude values for both fascicles are located on the lower electrode rows, i.e. on the “tips” of the array, which might indicate that the array was not perfectly inserted in the centre of the nerve. For the CF MEA, due to its geometrical conformation, no vertical discrimination is expected.

Objective assessment of fascicle discrimination power was made using the distance metric. There was a significant (p < 0.05) difference between mean T/P fascicle CoM distance estimated with different techniques (*p* = 0.00096, repeated measures ANOVA, within-subject factor). The mean fascicle distance estimated by each technique was 402 ± 30 μm for EIT, 414 ± 123 μm for CF MEA, 103 ± 51 μm for SS MEA, and 625 ± 17 μm for MicroCT. Normalised with respect to a nominal nerve diameter of 1400 μm, these values correspond respectively to 28.7 ± 2.1% for EIT, 29.6 ± 8.8% for CF MEA, 7.4 ± 3.6% for SS MEA, and 44.6 ± 1.2% for MicroCT. Individual comparisons between techniques reported significant (p < 0.05) difference between the distance estimated with SS probes and all other techniques, no significant difference between CF and EIT/MicroCT, but significant difference between EIT and MicroCT (EIT vs CF *p* = 0.929, EIT vs SS *p* = 0.0014, EIT vs MicroCT *p* = 0.0085, CF vs SS *p* = 0.0385, CF vs MicroCT *p* = 0.1854, SS vs MicroCT *p* = 0.0013, paired T-test comparisons). The individual t-test against zero mean for each technique showed significant (p < 0.05) discrimination power for each technique except for SS probes (EIT *p* = 0.00019, CF *p* = 0.028, SS *p* = 0.12, MicroCT *p* = 0.0000032, individual t-tests).

### 3.3 Analysis of scatter in clusterized fascicle data and comparison of superimposed results

**Fig. 8:**
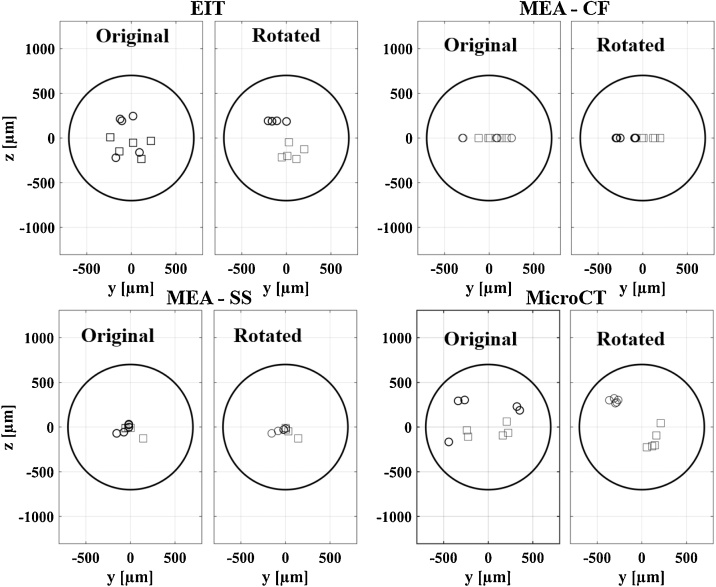
Clusterization procedure. Tibial (squares) and peroneal (circles) data is rotated with an individual angle of rotation for each nerve and each technique in order to minimize scatter of data among the same fascicle groups.

Assessment of internal scatter in fascicle clusters was performed after applying the clusterization procedure described in Section 2.7 (Fig. 8). Post-clusterization mean scatter was 64 μm for EIT, 49 μm for CF MEA, 47 μm for SS MEA, and 58 μm for MicroCT – very similar for all techniques and corresponding on average to 3.9% of a nominal nerve diameter of 1400 μm. Average rotation angles were 22° for EIT, 108° for CF MEA, 62° for SS MEA, and 29° for MicroCT.

**Fig. 9:**
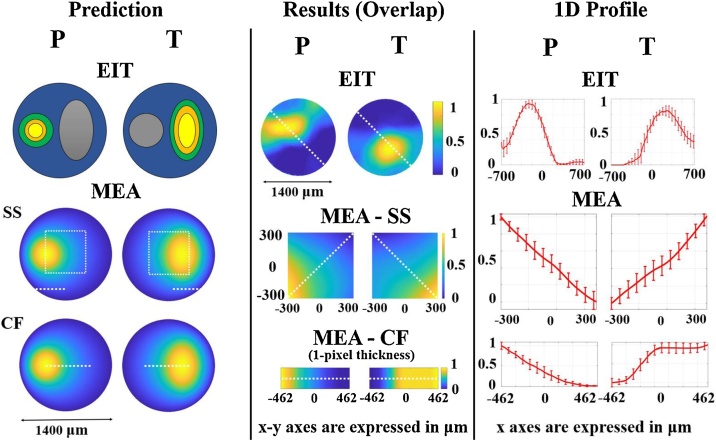
Comparison between prediction and results for EIT and MEA data. Left panel: predicted conductivity (EIT) and voltage (MEA) distributions over the nerve cross-section in response to evoked fascicular activity. Middle panel: average EIT and MEA images generated from overlaying the entire dataset (N = 5) for each technique. Right panel: 1D profiles ± 1SE extracted from EIT and MEA images in middle panel. SE is computed over individual images from each nerve (*N* = 5) in the dataset. In left panel MEA, white dotted lines indicate the area covered by SS and CF probe electrodes and imaged in middle panel. In middle panel EIT and MEA, white dotted lines indicate the 1D profile analysed in the right panel.

A qualitative comparison between predicted and resulting spatial signal distribution was performed for EIT/MEA images obtained by overlaying data collected from each nerve: predictions and results were found to be in good general agreement (Fig. 9).

## 4 Discussion

### 4.1 Summary of results

It was possible to record reproducible and significant changes related to neural activity with both EIT and MEA probes as both impedance changes and CAPs are significantly higher than background noise. On average, CAP recordings from CF probes had higher peak amplitudes compared to SS probes (CF ∼700 μV vs SS ∼300 μV, +130%); however, CF probes also showed much higher background noise (CF ∼6.3 μV vs SS ∼1.7 μV, +270%), thus leading to an overall lower SNR. CAP analysis also showed a high variability in peak amplitude recorded for different nerves and fascicles, resulting in a high SE around the mean values reported above for both types of probes. We attribute this variability to factors that, despite our best efforts at standardising the procedure, influenced the outcome of probe insertion (and thus electrical contact with the nerve fibres) such as:

• Variability in epineurium and perineurium thickness between nerves.
• Variability in relative positioning of the fascicles, i.e. one fascicle might be more or less accessible to the probe than the other.

The distance metric we defined allowed us to evaluate the capability of each technique to discriminate fascicle position. As expected, our reference technique of MicroCT returned the highest and most realistic value of ∼600 μm. This is appropriate considering an average diameter of 1400 μm for the rat sciatic nerve and that each fascicle occupies roughly half of the entire nerve cross-section and confirms the quality of this technique as gold standard. The most interesting result from this analysis is the average distance between fascicles reported for EIT and the CF probes, ∼400 μm for both methods. This value is approximately one-third lower than MicroCT; our statistical analysis found EIT, but not CF, to be significantly different from MicroCT. However, CF data itself had no significant difference from EIT, suggesting that both techniques have good source localisation power and that similarity of CF to MicroCT is most likely only due to its standard deviation, which is higher than EIT. Discrimination power for the SS MEAs was low and with no statistical significance. However, this result does not necessarily imply a lack of source localisation power for SS MEAs in general but can be interpreted as the consequence of trying to insert a probe with multiple shanks with non-negligible width into the cross-section of the nerve. The most likely explanation and our main hypothesis is that during insertion, the width of the shanks led to driving the nerve tissue apart, away from the electrodes. In such a condition, recording compound activity would still be possible due to the conductivity of the surrounding extracellular environment, thus explaining the recorded CAPs. However, the recorded field would lack the spatial resolution required for source localisation.

The assessment of internal variability in fascicle CoM position within clusters returned very similar values after performing our clusterization procedure, with the values for each technique contained in a range of ∼45–65 μm. The average rotation angles required to achieve minimum scatter within each technique are themselves a result, as they can be interpreted as an indication of the amount of deformation associated with each technique which needs to be corrected by the clusterization procedure. As expected, the lowest rotation angles (∼20–30°) are associated with the non-invasive techniques of EIT and MicroCT, and the highest angles (∼60–110°) with the invasive probes. The average angle of rotation is higher for the SS probe compared to CF, which is counterintuitive considering that the CF probes showed better performance in source localisation. However, Fig. 8 shows that having all the CF datapoints lying on the Y-axis has the effect of producing only 0° or 180° rotations during clusterization. As such, the average rotation angle for CF probes seems to be overestimated and is probably equal or lower to that of SS MEAs.

Superimposition of data from all five nerves after the clusterization procedure allowed for a comparison between predicted and achieved results. In Fig. 9, the spatial distribution of superimposed EIT data from this study is in qualitative agreement with prediction, which in turn is based on typical nerve EIT images from previous studies (Aristovich et al., 2018; Ravagli et al., 2019, 2020). Peak fascicle activation is clearly distinguishable from the 1D profiles analysed along the cross-section of the nerve. For MEA probes, the result comparison should consider the nature of the signal we investigated in this study, the CAP. During electrically-evoked CAPs, all the fibres from the same fascicle fire at the same time and act as a group of densely-packed individual current sources. Volume conduction in the extracellular space then gives rise to an averaged signal which resembles the individual AP (Fig. 6) but is distributed across a wide area of the nerve's cross-section. Even disregarding volume conduction, voltage signal would be highly homogeneous across most of the fascicle as electrical stimulation implies that the fibres fire with high synchronisation. Our FEM simulation where the fascicle acts as a current source shows a spatial spread of the voltage signal of ∼250 μm, similar to the minimal reported spatial distribution of LFPs in brain. This is a reasonable result considering that, as mentioned in the Introduction, CAPs and LFPs share some similarities. Superimposed experimental results for SS and CF probe recordings are in visual agreement with the simulation results (Fig. 9, MEA Prediction and Results): both show a smooth spatial distribution of voltage and suggest that our modelling of the involved biophysics is realistic. Both MEA recordings and EIT images resulted in a smooth spatial distribution of the signal of interest, voltage and conductivity changes, respectively. This is an effect of the physics of volume conduction, which involves the creation of a smooth and widely distributed electrical field even in response to the activation of a source such as the fascicle, which has very clearly defined boundaries.

### 4.2 Technical issues and limitations of this study

In the present study, EIT was performed at the level of the epineurium, i.e. non-invasively for the nerve, using electrodes placed radially around the nerve on a cuff, and MEA recordings are performed inside the nerve as passive voltage recordings. A more direct comparison would be between EIT performed with the cuff electrodes and with the MEA electrodes. However, performing nerve EIT directly on the MEA probes is prevented by the high impedance of their electrodes: injecting EIT current with sufficiently high amplitude to achieve minimum SNR for image reconstruction would be damaging to the nerve tissue. A possible solution, theoretically, would be to shunt individual electrodes together to lower impedance on injecting pairs (Faulkner, 2019); however, this solution is unachievable with the probes used in this work due to the high ratio between average electrode impedance and total number of electrodes.

One of the main limitations of this study concerns the computation of CoMs for MEA probes directly from recorded voltages and electrode coordinates rather than using recorded data to perform ISA and compute CoM from the reconstructed voltage distribution. This is a simplified methodology and conceptually it poses the risk of underestimating the distance of the CoM from the centre, since the recording electrodes do not span the full diameter of the nerve (∼1400 μm) but stop approximately halfway at ∼600 μm and ∼900 μm for the SS and CF probes, respectively. However, the maximum possible error introduced by this simplification is strongly limited by the fact that in the rat sciatic nerve, the tibial and peroneal fascicles each occupy approximately half of the total diameter. For this reason, CoMs location cannot be reasonably located more than ∼300–350 μm from the centre, a distance which is very close to the maximum extension of the SS probe (±300 μm) and inside the reach of the CF probe (±472 μm); thus, the error induced by our CoM computation method would be, even with some variability, statistically low. Our reasoning is supported by the fact that both MicroCT, our reference technique, and EIT, which images the whole 1400 μm diameter, found CoM locations not further away from the centre than 300–350 μm. More so, even with this limitation, CF probes were found to be in agreement with EIT and MicroCT (see Results section). For a nerve with a larger quantity of smaller fascicles like the vagus nerve, switching to ISA-based CoM computation for MEA probes will be required.

In the present work, CAPs were collected and analysed independently from EIT. In future studies, CAPs will be extracted directly from the lower frequency bandwidth of EIT recordings as done in previous nerve EIT work (Aristovich et al., 2018) to reduce data collection times.

Due to the presence of large stimulation artefacts in some CF and NN recordings, a small part of the dataset had to be collected from CAP negative peaks. The use of this procedure is not likely to impact the outcome of the study, since SNR is mainly determined by probe placement in relation to the fascicle, which influences CAP amplitude, and by number of averages, which influences residual noise level. More so, this procedure has only been employed for 20% of the recordings, and in nerves where CAP is fully separated from stimulation artefacts, the negative peak is usually similar in amplitude to the positive peak (Fig. 6).

The use of penetrating probes in the nerves raises a legitimate concern about the validity of the recorded data as one or both of the MEAs could be mechanically damaging to the tissue. More so, insertion of the first probe could have an influence on the quality of the data collected with the second probe. However, the data collected in this study appears to be reliable based on the following criteria:

• For every probe, more than 80% of recording sites were functional at the time of recording and non-functional sites were excluded from our analysis.
• CAPs were successfully recorded in all instances with both types of probes, indicating healthy neural tissue at all times.
• Recording CAPs with the first probe used on the nerve, i.e. the CF MEA, was a requirement for proceeding with SS MEA insertion. This requirement ensured that SS MEA recordings were performed only in case of unsubstantial tissue damage by the CF MEAs.
• MicroCT scans of the nerve taken as reference were also assessed and no significant damage was detected; although, nerve tissue displacement caused by probe insertion could be partially reversible after probe removal due to the elasticity of the tissue.

For this work two types of probes were chosen to represent different MEA technologies, silicon and CF. Both types of probes had advantages and drawbacks:

• Silicon probes came with the advantage of built-in bidimensional sampling in the form of the 4 × 4 grid of electrodes, but the large width of the shanks made insertion into the nerve more difficult and most likely contributed to the lack of success we had in fascicle localisation with this technology.
• With CF MEAs, insertion into the nerve was easier due to the extremely small diameter of the individual fibres; however, the geometric arrangement only allowed measurements at fixed depth.

The use of different electrode geometries for CF and SS technologies led to the presence of some limitations: different CoM calculation methods had to be adopted; MEAs had limited recording volumes compared to the diameter of the nerve; comparison of source localisation for 1D CF probes and 2D SS probes required a scaling factor assumption of √2. Our choice of scaling factor was driven by the logic that any CoM measured by the CF probes only corresponds to the projection over the lateral axis of a CoM in 2D space, for which 45° is the average angular location, corresponding to a real CoM distance from the centre which is √2 times larger than the CF-measured values. As explained in Experimental Design, the probe designs we adopted for this study were those available from the manufacturers but with some of the geometrical parameters customised for our application (mainly the length of the shanks or carbon fibres). The main focus of future work will be the use of enhanced probe designs removing the need for geometrical assumptions.

The order of the recordings performed in each experiment, corresponding to EIT first, then CF MEA, then SS MEA, was fixed by design. While this choice introduces the risk of a bias in the results, our assessment was that the benefit of proceeding in an order of increasing invasiveness was greater in comparison. In future studies, additional testing may involve randomisation, or comparison of data recorded from one specific type of probe in stand-alone experiments vs recordings performed with the same type of probe with others.

In this study, *N* = 5 sciatic nerves were tested from three different animals, meaning that in two animals both left and right nerves were subject to our measurements. While it is true that nerves and fascicles from the two sides of the animal may share similar size, influence of within-animal correlation in the study is limited by the following factors: i) physiological variability may be present inside the animal, reducing the similarity between left/right sides, and ii) all animals were chosen to be in a narrow weight range (400–550 g) meaning that overall animal size, and consequently nerve size, were very similar across animals. Combination of these factors suggested a very small difference between inter-animals and intra-animals variability.

Our study focused on recording evoked CAPs, but previous works in literature also reported recording of single- or multi-unit activity in nerves using microelectrodes. Unit activity is associated with individual or small groups of fibres; however, it is usually recorded during spontaneous firing. During evoked activity, all fibres from the same fascicle or nerve are firing at the same time with strong synchronisation, and thus the recorded signal is not an expression of individual fibre activity anymore but is dominated by a field potential which is the sum of all fibre activity. In this work, electrical stimulation of individual fascicles was required to create localised sources of neural traffic and as such CAPs were chosen as the signal of interest for our analysis.

Lastly, one of this study's limitations concerns the simplicity of the experimental model. The rat sciatic nerve was chosen for its clear fascicular organisation, with two major fascicles of comparable diameter that do not intermingle between the stimulation and recording sites. However, the complex peripheral nerves such as the vagus nerve have multifascicular organisation. A lot of evidence points to a high degree of somatotopic organisation of nerve fibres in such nerves (Bäumer et al., 2015; Stewart, 2003; Zill et al., 1980) – fibres supplying one organ/tissue are arranged in one or several fascicles running in close proximity to one another. In the case of the vagus nerve in large mammals, the distance between the target organ and the recording/stimulation site can reach tens of cm, and some degree of fascicle intermingling is present. In our study, both MEA and fast neural EIT provided localised images of neuronal depolarisation, but in multifascicular nerves these images will not necessarily be restricted to individual fascicles. In application to multifascicular nerves, these techniques are expected to identify the CoM of evoked or spontaneous organ/tissue-specific activity. Given that the main purpose of imaging fascicular activity non-invasively is the subsequent stimulation/block of the identified fascicle(s) to achieve specificity of neuromodulation, EIT provides a unique solution for this purpose even in the case of complex multifascicular nerves. Fascicle identification in multifascicular nerves using MEA probes would need further development of the MEA probe design, which is discussed in Section 4.4.

### 4.3 Answers to questions in 1.4

As a result of our work, we can now answer the questions we posed at the beginning of this manuscript:

a) Is it possible to record fascicular evoked CAPs with multi-electrode probes in rat sciatic peripheral nerves, together with EIT?
  Yes. Both MEA technologies we tested in this study were able to record evoked compound activity from within nerves with amplitude in the range of hundreds of μV, all after performing successful EIT recordings and image reconstruction.
b) Which methodological details are important for proper insertion of multi-electrode probes in the rat sciatic nerve?
  We found the following technical features to be important for achieving probe insertion:
  • Tip sharpening with procedures developed by probe manufacturers.
  • Enzymatic digestion of the epineurium facilitates the insertion of the probes; however, the insertion of relatively wide and thick shanks (100 × 50 μm in case of SS MEA) is still challenging.
  • For transversal insertion of a MEA probe, the mechanical stabilisation of the nerve and gentle distention in longitudinal direction is necessary.
c) How does source localisation on the nerve cross-section compare between the invasive multi-electrode probes and fast neural EIT? Which technique gives the highest spatial separation between fascicles?

The highest localisation power excluding the reference technique of MicroCT is achieved by EIT and CF probes. CF probes showed localisation power lower but comparable to MicroCT, and EIT localisation power was similar to CF. SS-based probes adopted in this study failed to discriminate fascicle location on the nerve cross-section surface.

### 4.4 Future work

Future stages of this work will revolve around the development of improved probe designs to address the current technical limits. As mentioned in Section 2.1, ideal configuration would have been a square grid of electrodes placed along the cross-section of the nerve, or a 3D structure allowing penetration at different depths like the USEA probes. These options do not necessarily require improvement in manufacturing technologies but could be achieved by improvement of geometrical arrangements. For example:

• The CF MEA design we used could be extended by stacking several rows of fibres with different length along the longitudinal axis of the nerve in order to build a structure similar to the Utah array and resolve the issue of fixed depth. One critical issue of this design would be to ensure penetration with the shorter fibres after the longer ones have already entered the nerve. Also, the different rows should be stacked at a distance short enough to guarantee that no fascicle merging or reorganisation happens along the recording area. This would ensure that recorded data from different rows can be stacked to compose a reliable 2D cross-sectional image.
• Silicon probes with multiple shanks, such as the one in this study, could be aligned on parallel planes oriented along the longitudinal direction of the nerve (i.e. the shank's plane would be lying on the X-Z plane from Fig. 5). This change in orientation would help the shanks slide inside the nerve along the fibres with a reduction in damage and deformation of the tissue. The main concern with this type of probe would be the non-trivial stacking of at least two multi-shank probes and the design of proper electrical connections and housing mechanics. More so, shank width should be reduced as much as possible, even though this may require a reduction in electrode diameter.

For both configurations above, a grid of 4 × 4 electrode would suffice, provided it covers at least 80% of the nerve's diameter, but a higher electrode count would of course be desirable (e.g. 8 × 8, 16 × 16). Our current method for computing CoMs from MEA recordings will be replaced with ISA for improved accuracy.

Future stages of the work will also involve switching from recordings of electrically-evoked activity toward recording physiological activity. Both MEA and EIT will suffer in terms of SNR from moving to recording of spontaneous activity due to the loss of coherent averaging as a technique for noise reduction. In addition to this, the signals recorded in this study originated from larger myelinated fibres, while in autonomic nerves, the majority of fibres are slow conducting C fibres, which may render the signals of interest vanishingly small. We predict the following strategies, alone or combined, will offer potential for spontaneous recordings:

• Replacement of electrical stimulation with autonomic stimulation, evoked by drugs or physical manoeuvres.
• Use of physiological signals as triggers for coherent averaging (e.g. heartbeat, breathing, gastric signals for vagus nerve).
• Longer averaging times.
• Advanced signal processing methods and use of different protocols, such as the box-car paradigm where signal changes are imaged between two windows: baseline and increased activity.

In future work, both MEA and EIT will be assessed in the vagus nerve of large animals (pigs or sheep). Following that step, EIT may be translated to human vagus nerve studies as the only non-nerve damaging method.

## Authors contribution

**Enrico Ravagli:** Methodology, Software, Formal analysis, Investigation, Data Curation, Writing - Original and Revised Drafts; **Svetlana Mastitskaya:** Methodology, Investigation, Writing - Original and Revised Drafts; **Nicole Thompson:** Methodology, Investigation, Data Curation, Writing - Review & Editing, Visualization; **Elissa J Welle:** Methodology, Resources, Writing - Review & Editing; **Cynthia A Chestek:** Methodology, Resources, Writing - Review & Editing; **Kirill Aristovich:** Conceptualization, Methodology, Formal analysis, Writing - Review & Editing, Supervision; **David Holder:** Conceptualization, Writing - Review & Editing, Supervision, Funding acquisition.
