# Supplementary material for: Fascicle localisation within peripheral nerves through evoked activity recordings: A comparison between electrical impedance tomography and multi-electrode arrays
Source: J Neurosci Methods. 2021 Jul 1;358:109140. doi: 10.1016/j.jneumeth.2021.109140 (PMC8249910; doi:10.1016/j.jneumeth.2021.109140)
Supplement: Supplementary file 1 [file mmc1.pdf]

Supplemental Table S.T1 – Centre-of-Mass (CoM) coordinates for every nerve analysed and for each measurement technique. Values are reported in microns according to the coordinates system defined in Figure 5 of the main manuscript. Hind leg for every animal is marked as L/R for left/right. Fascicle from which neural activity is evoked is marked as T/P for Tibial/Peroneal. EIT = Electrical Impedance Tomography; CF = carbon fibre; SS = silicon shank.

				EIT		CF	SS		MicroCT	
**Nerve**	**Animal**	**Hind leg**	**Fascicle**	**Y(μm)**	**Z(μm)**	**Y(μm)**	**Y(μm)**	**Z(μm)**	**Y(μm)**	**Z(μm)**
**#1**	#1	L	T	112	-235	138	143	-126	163	-94
			P	-106	192	-300	-154	-70	-265	303
**#2**	#2	L	T	-134	-149	196	3	-9	207	62
			P	19	245	-290	-74	-57	-337	293
**#3**		R	T	20	-52	-118	-56	-14	224	-65
			P	-126	213	252	-19	26	-442	-166
**#4**	#3	L	T	-237	8	11	-27	-3	-226	-108
			P	91	-161	92	-16	0	321	230
**#5**		R	T	220	-33	-9	-56	-9	-240	-36
			P	-174	-220	81	-18	33	352	187

Supplemental Table S.T2 – Values of the distance between fascicles computed for each nerve in the dataset according to Section 2.7 of the main document. Values are expressed in microns, with mean ± 1 standard deviation (SD) for each group. Values for the CF group are corrected as explained in the main manuscript.

Nerve	EIT (μm)	CF (corrected) (μm)	SS (μm)	MicroCT (μm)
**#1**	480	619	303	583
**#2**	423	688	91	591
**#3**	303	523	54	674
**#4**	369	113	11	643
**#5**	436	126	57	633
**Mean±SD**	402±68	414±275	103±115	625±38

Supplemental Table S.T3 – Updated CoM values from supplemental Table S.1 after performing the clusterization procedure reported in Section 2.7 of the main manuscript. For each nerve, the rotation angle that minimizes intra-group scatter is reported.

		EIT			CF		SS			MicroCT		
**Nerve**	**Fasc.**	**Y(μm)**	**Z(μm)**	**Rot. (°)**	**Y(μm)**	**Rot. °**	**Y(μm)**	**Z(μm)**	**Rot. (°)**	**Y(μm)**	**Z(μm)**	**Rot. (°)**
**#1**	T	112	-235	0	138	0	143	-126	0	163	-94	0
	P	-106	192		-300		-154	-70		-265	303	
**#2**	T	11	-200	45	196	0	2	-10	-10	212	43	-5
	P	-160	187		-290		-83	-43		-310	321	
**#3**	T	29	-48	10	118	180	32	-47	110	56	-226	-60
	P	-161	188		-252		-18	-26		-365	300	
**#4**	T	201	-126	150	-11	180	5	-26	95	145	-203	100
	P	2	185		-92		2	-16		-282	276	
**#5**	T	-52	-216	-95	9	180	32	-47	115	116	-213	110
	P	-204	192		-81		-22	-30		-297	267	

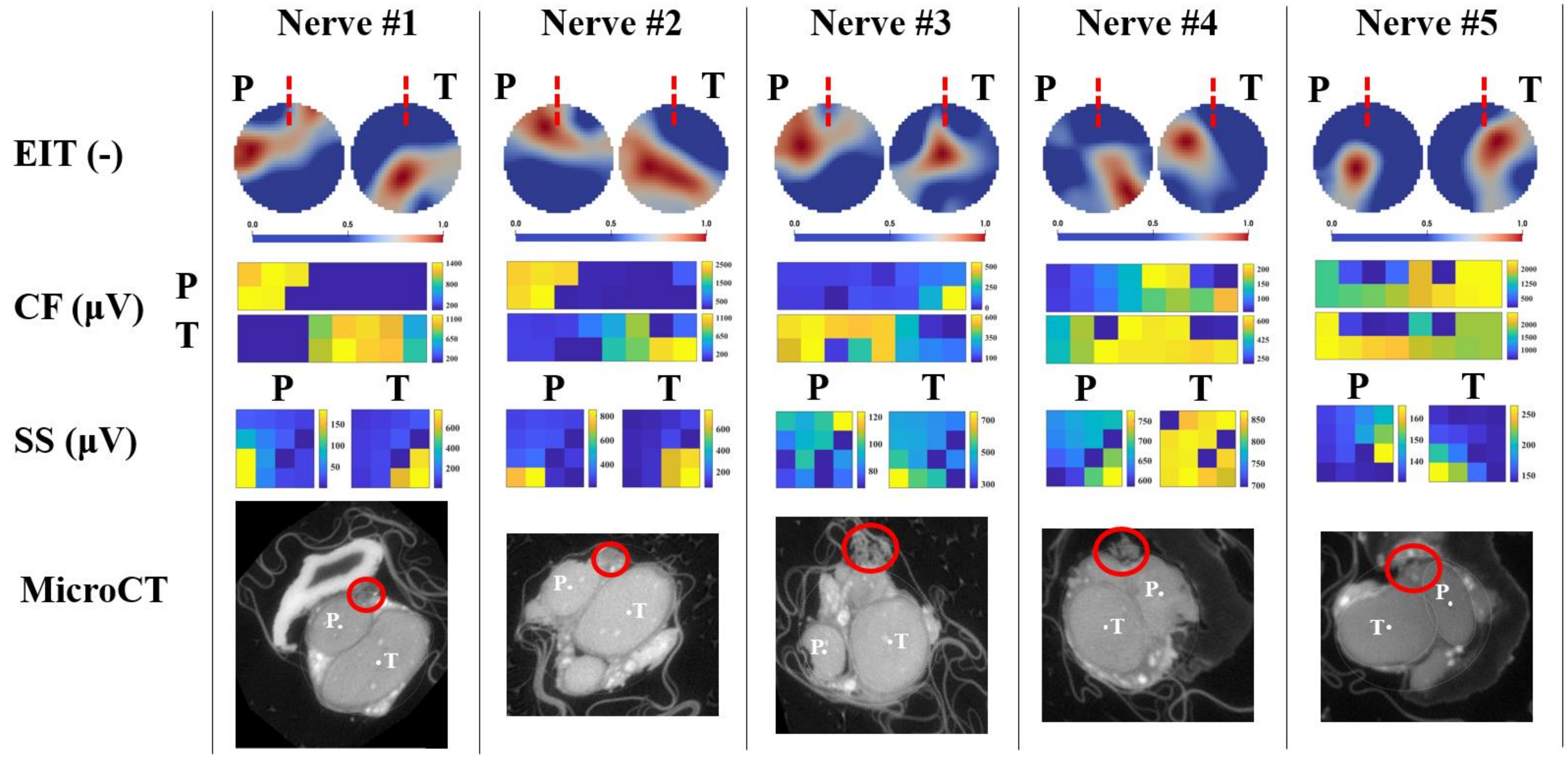

Supplemental Figure S.1 – Images from EIT, MEA and microCT from all five nerves. EIT: the individual images of evoked fascicular activity reconstructed from EIT recordings for the tibial (T) and peroneal (P) fascicles, respectively. The range of values for every image was normalised between 0 and 1 and the rainbow colour scale is showing the top 50% of colour for each image i.e. full width at half maximum (FWHM) intensity scaling. Cuff opening position is marked by dotted red lines. CF: voltage recordings from carbon fibre probes expressed in µV. Each pixel correspond to a recording site in the 8x2 electrode configuration described in the main document. SS: voltage recordings from silicon shanks probes expressed in µV. Each pixel correspond to a recording site in the 4x4 electrode configuration described in the main document. MicroCT: representative slice of the microCT scan of the nerve in the area corresponding to EIT/MEA recordings with fascicles clearly visible. Snippets of the silk thread marking the EIT cuff opening area are highlighted with red circles. All EIT/microCT images were rotated so that the electrode cuff opening was at 12 o'clock. The external boundaries of the nerve were fitted to a circular profile after rigid deformation. Each fascicle was fitted into an ellipsoid profile (dashed white line) with a CoM (white dot in the middle) which was further used as a ground truth coordinate in the study.
